# Acupuncture for the treatment of radiation-induced xerostomia among patients with cancer: A protocol for a systematic review and meta-analysis

**DOI:** 10.1097/MD.0000000000020658

**Published:** 2020-06-12

**Authors:** Rumeng Chen, Yang Gao, Xianliang Qiu, Peipei Hong, Dongqi Zhou, Qiu Chen

**Affiliations:** Hospital of Chengdu University of Traditional Chinese Medicine, Sichuan, China.

**Keywords:** acupuncture, cancer, protocol, radiation, systematic review and meta-analysis, xerostomia

## Abstract

**Background:**

With the number of cancer patients growing, radiotherapy and chemotherapy have been a necessary treatment. Unfortunately, there are many side effects after radiation and chemotherapy, one of which is xerostomia that always harasses patients. Although there are many ways of treatment of xerostomia, they have many disadvantages. With the rare side effects and the excellent effect, acupuncture has been widely applied to dry mouth after radiotherapy, but it has not been recognized as the standard treatment. Because acupuncture prescription is mostly different and the sample size of studies is small, we need more high-quality meta-analysis to provide relatively reliable evidence for the treatment of radiation-induced xerostomia. The objective of this study is to assess the curative effect of acupuncture treatment of cancer patients after radiotherapy and provide more reliable evidence for acupuncture treatment of xerostomia after radiotherapy for cancer patients.

**Methods:**

We will search the following databases: CENTRAL (The Cochrane Central Register of Controlled Trials), MEDLINE, EMBASE, PubMed, CNKI (China National Knowledge Infrastructure), VIP (China Science and Technology Journal Database), Wan Fang Data Knowledge Service Platform. At any rate, 2 review authors will assess all randomized controlled trials (RCTs), seemingly conformance to the inclusion criteria, to confirm qualification, determine the risk of bias and extract data using a running data extraction form. The revolution of disagreements is a discussion. We will use the approach recommended by Cochrane reviews to assess the bias in studies. Risk ratios (RR) and 95% confidence intervals (CIs) will be used to assess the treatment effects of an intervention for dichotomous results. We will use mean differences (MD) and standard deviation (SD) to aggregate the data of every trial for continuous results. The heterogeneity test of Cochran and quantification of the *I*^2^ statistic will be used to assess the variation of treatment effects. Only if there are studies of semblable comparisons reporting the same results, we will conduct a meta-analysis.

**Results:**

From the study, we will evaluate the efficacy of acupuncture for xerostomia patients who has cancer and been treated by radiation.

**Conclusion:**

The conclusion of this study will be the evidence, which can ensure the efficacy of acupuncture for cancer patients with radiation-evoked xerostomia among and provide guidance for the treatment of xerostomia.

**INPLASY registration number:**

INPLASY202040211.

## 1 Introduction

With approximately 18.1 million new cases and 9.5 million cancer-related deaths in 2018, cancer has long been one of the primary causes of morbidity and mortality worldwide.^[1]^ Cancer is usually treated with radiation, either as a monotherapy or a combination of chemotherapy and surgery. Xerosis is regarded as one of the side effect of radiotherapy^[2]^ and chemotherapy^[3]^ for cancer. The seriousness of xerostomia depends on the total exposure of the sialaden to radiation, or the amounts of chemotherapy drugs applied.^[3]^ When salivary glands are in the irradiation range, irreversible salivary gland damage occurs in 63% to 93% of patients.^[4]^ Salivary gland damaged results in reduced salivary secretion, which leads to the change such as dry mouth, taste changes, difficulty with chewing, swallowing, speaking, and an increased risk of dental disease.^[4]^ In general, too little saliva and associated dry mouth can cause a significant reduction in quality of life.

Dry mouth disease can be treated in many ways. However, we still not have an answer to which methods are the most effective and appropriate since there are different characteristics in different approaches. Assessing the residual secreting capacity of the salivary glands is essential. Taste and mechanical stimuli, such as peppermint or sugarless gum, are supposed to be involved in treating while the salivary gland can still be stimulated. Otherwise, systemic cholinergic agonists should be considered. Pilocarpine, as a famous cholinergic agonist, can stimulate the flow of saliva in both healthy volunteers and dry mouths patients.^[5]^ Unfortunately, this pill may also lead to some side-effects such as headaches, nausea, vomiting, and frequent urination etc.^[6]^ Using palliative mouth care products, including mouthwash, oral gel, and saliva replacements can alleviate care products.^[7]^ But palliative mouth products also have some restrictions. They are short-lived, lack protective effects, and the saliva may expel from the mouth as swallowing.^[3]^ As a consequence of these limitations, complementary and alternative medicine (CAM) is increasingly popular among patients with dry mouth,^[8]^ and one of the most extensively used CAM treatments is acupuncture.

Acupuncture has been used as a traditional treatment in traditional Chinese medicine for at least 2500 years and has been popular around the world since the 1970s. According to data from World Health Organization (WHO), acupuncture is used in at least 103 countries, 29 of which have developed regulations for providers, and 18 of which have allowed third-party coverage.^[9]^ Acupuncture has been shown to treat and relieve 64 symptoms in 2003.^[10]^ The acupuncture is widely used to manage the symptoms of cancer at present. The reason why acupuncture is a good selection for many cancer patients and cancer survivors is that it can treat various types of cancer-related symptoms at the same time with an exceedingly low risk of adverse reaction (less than once per 20,000 people).^[11]^ Some studies have investigated the efficacy of acupuncture on dry mouth.^[12,13]^ According to reports, acupuncture and electrical stimulation have biological and clinical reasonability in treating dryness of the mouth.^[14–16]^ Although some studies have shown positive effects, a meta-analysis about the efficacy of acupuncture in the treatment of salivary secretion or dry mouth after radiotherapy for cancer is still lacking.

Acupuncture relieves many symptoms by inserting wonderful, solid needles into the skin or at sites under the dermis.^[11]^ There are several hypotheses about how acupuncture increases saliva production. Acupuncture stimulates the sympathetic and parasympathetic systems by activating neurons.^[17,18]^ Besides, acupuncture promotes the neuropeptides, such as the genetic peptide of vasodilator calcitonin, have an anti-inflammatory effect, and nutritional efficacy on the sialaden, and increase blood flow to the acinus.^[17,18]^ Moreover, acupuncture produces physiologic efficiency, such as stimulating the vegetative nervous system and increasing peripheric blood flow, which in reverse stimulates the secretion of saliva.^[14]^ It also has been suggested that acupuncture can immediately increase the blood flow near the salivary gland, thereby increasing the secretion of the salivary glands.^[19]^

With the number of cancer patients growing, radiotherapy and chemotherapy have been a necessary treatment. Unfortunately, there are many side effects after radiation and chemotherapy, one of which is xerostomia that always harasses patients. Although there are many ways of treatment of xerostomia, they have many disadvantages. With the rare side effects and the excellent effect, acupuncture has been widely applied to dry mouth after radiotherapy, but it has not been recognized as the standard treatment. Because of different acupuncture prescriptions and the small sample size, we need more high-quality meta-analysis to provide relatively reliable evidence for the treatment of radiation-evoked xerostomia. The objective of this study is to assess the curative effect of acupuncture treatment of cancer patients after radiotherapy and provide more reliable evidence for acupuncture treatment of xerostomia after radiotherapy for cancer patients.

## 2 Methods

### 2.1 Study registration

The present protocol has been registered in the INPLASY (registration number INPLASY202040211; DOI:10.37766/inplasy2020.4.0211), which can be available on https://inplasy.com/inplasy-2020-4-0211/. The article will be reported to conform to the Preferred Reporting Items for Systematic Review and Meta-Analysis Protocols (PRISMA-P)^[20]^ statement.

### 2.2 Inclusion criteria for study selection

#### 2.2.1 Type of studies

Randomized controlled trials (RCTs), due to the instability of the conditions being reviewed, we will not include studies using cross-design, case-control studies, or studies without controls. No language restrictions.

The exclusion criteria will be the following:

Full article not available.

The coordinated intervention was unbalanced between the 2 groups.

Studies used noninvasive acupuncture.

#### 2.2.2 Type of participants

To evaluate the efficacy of acupuncture for xerostomia reduced by radiation among cancer patients, we will include any patients with xerostomia caused by cancer radiotherapy. Non-radiation and non-cancer related xerostomia will not be contained.

#### 2.2.3 Type of interventions

We will only contain invasive acupuncture studies for evaluating the therapeutic effect of acupuncture. Changes: meeting, frequency, duration, doctor's background and details of the acupuncture point, depth, length of stay, number of needles are admissible. The intervention and control groups can be placebo or routine care groups. Invasive acupuncture is defined as piercing the skin with a needle. On the contrary, noninvasive acupuncture refers to acupuncture that does not use needles or needles do not stab the skin, for example, laser acupuncture. Combined interventions will be permitted, such as Chinese traditional medicine, but they must be equilibrated between the 2 groups.

#### 2.2.4 Type of outcome measurements

There are 2 primary and 3 secondary outcomes.

Primary outcomes:

(1) Objective: salivary gland flow rate, salivary gland scintigraphy, or salivary gland functional magnetic resonance imaging.
(2) Subjective: subjective measures of patients’ self-reported scores (the visual analog scale [VAS], the dry mouth scale, or quality of life).

Secondary outcomes:

(1) Patient satisfaction with the treatment.
(2) The validity of time.
(3) Safety indicators: the incidence of adverse reactions and the proportion of patients with abscission.

### 2.3 Search strategy

The following databases will be searched: CENTRAL, MEDLINE, EMBASE, PubMed, CNKI, VIP, Wan Fang Data Knowledge Service Platform. As a part of the hand-searching programme of the Cochrane Collaboration, hand-searching carried out is incorporated in the search. The CENTRAL database will have been included the references of hand-searching. To determine current and completed trials, we will search the meta Register of Controlled Clinical Trials and ClinicalTrials.gov. No language restrictions. For further trials, we will look up relevant review articles and all articles. Reference of the extracted studies also will be searched by hand for further relevant articles.

The search strategy will be the following group terms (Table 1).

**Table 1:**
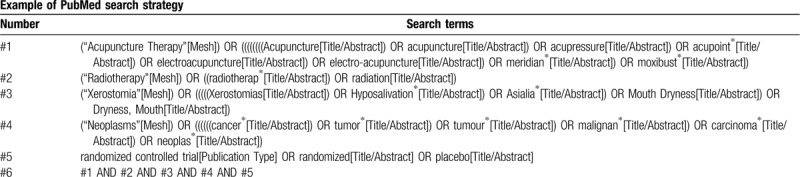
PubMed search strategy.

### 2.4 Selection of studies

The searched results will be screened by >1 review authors to confirm possible included studies. It is necessary that obtaining paper copies of all trials which likely conform to the inclusion criteria or where there is inadequate information in the title or abstract or both to make a specific determination about qualification. All papers will be assessed by at least 2 review authors to determine which meet the inclusion criteria for this review. Any differences will be settled by discussion. According to the requirements of the members of The Cochrane Collaboration, Papers will be translated into English for non-English review.

**Figure 1:**
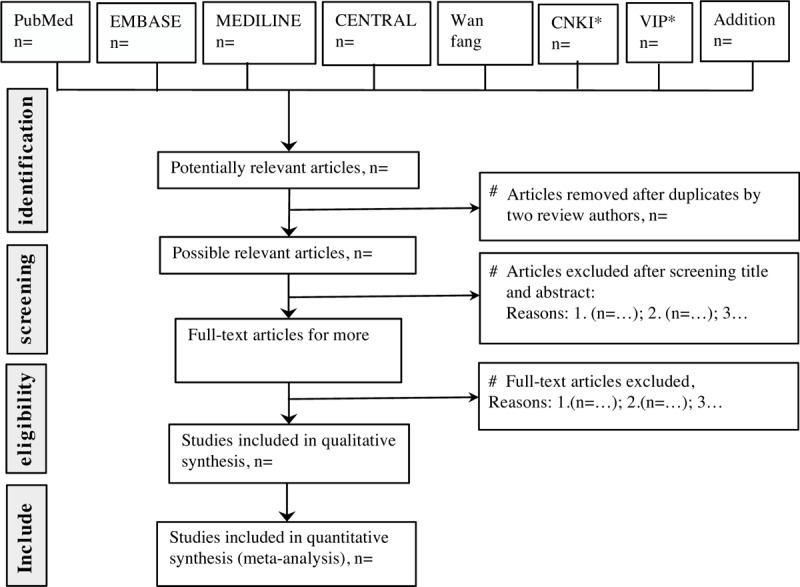
Study selection flow chart. CNKI^∗^—China National Knowledge Infrastructure; VIP^∗^—China Science and Technology Journal Database.

Flow diagram of literature search (Fig. 1).

### 2.5 Data extraction

At least 2 review authors will assess all RCTs, seemed to accord with the inclusion criteria, to affirm qualification, evaluate the risk of bias, and extract data by a running data extraction form. The revolution of differences will be a discussion. The following data (publication year; country; funding; the number of centers, patients-recruitment, dropouts, final-inclusion; Intervention; comparator; the volume of radiation-exposed; primary and secondary outcomes; duration measured) will be recorded.

We will try to connect with the authors of the studies when there are some ambiguous concerning aspects of trial design.

### 2.6 Assessment of risk of bias

We will use the approach recommended by Cochrane reviews to assess the risks of bias in studies.^[21]^ The 6 specific areas, such as sequence generation, hidden allocation, blinding, partial result data, selective result reporting, and “other bias,” will be solved by a 2-part tool. One or more particular entries will be included in a “Risk of bias” table for every domain. The first part of the device is that explaining what has occurred in the reported study. The second will judge that clause, which is obtained by answering a preassigned question about the correlation between the entrance and the study (low, high, unclear risk of biases were respectively described as low, high, ambiguous).

The tool will process the areas, sequence creation, grouping concealment, inadequate outcome data, and selective result reporting, using a single clause for every study. We will use blinding 2 clauses because patients and outcome assessors are needed to assess separately, particularly, patients will evaluate the results to the trial on themselves. According to the last domain, a single clause for studies will determine it as a whole.

Two authors will undertake the risk of bias assessment respectively when the data are extracted.

### 2.7 Measures of treatment effects

Risk ratios (RR) and 95% confidence intervals (CIs) will be used to evaluate the effect of an intervention for dichotomous results, such as xerostomia improved or not; we will use MD and SD to summarize the data of every trial for sequential results (e.g., mean saliva flow, VAS scores, self-rating report).

### 2.8 Assessment of heterogeneity

That examination of the point estimates and Fiducial inference on the forest plot will be used to evaluate heterogeneity. The heterogeneity test of Cochran and quantification of the *I*^2^ statistic will be used to assess the variation of treatment effects. If the *P*-value is <.1, heterogeneity will be with statistical significance. According to Cochrane Handbook for Systematic Reviews of Interventions Version 6.0, a rough guide to the interpretation of the *I*^2^ statistic is: 0% to 40% might not be significant, 30% to 60% may be on behalf of moderate heterogeneity, 50% to 90% may represent extensive heterogeneity, and 75% to 100% substantial heterogeneity.^[21]^

### 2.9 Assessment of reporting bias

In terms of the proposals on detection for funnel plots dissymmetry, publication bias will be assessed, if there have been abundant trials (over 10) in whichever meta-analysis. If asymmetry has been recognized. We will analyze the probable causes.

### 2.10 Data synthesis

Only if the studies of analogical comparisons reporting the same outcome measures, we will conduct a meta-analysis. We will combine RR for dichotomous data, and we will connect the average difference for successive data using the fixed effects model. If there are over 3 studies contained in whichever meta-analysis, we will use random effect models.

### 2.11 Sensitivity analysis

Sensitivity analysis based on the overall risk of bias will be undertaken if there has been enough research for each intervention and outcome.

### 2.12 Subgroup analysis

We intend to explore clinical heterogeneity by testing the issue of remission of xerostomia after acupuncture treatment. If sufficient research of each outcome and intervention has been conducted, we will aim to undertake a preliminary subgroup analysis of the different results of the remission of dry mouth.

### 2.13 Grading the quality of evidence

The main results of this review will be summarized using the Grading of Recommendations Assessment, Development and Evaluation (GRADE). We will evaluate the quality of the evidence by referring to the total risk of bias of the contained studies, the substantivity of the evidence, the inconformity of the results, the accuracy of the estimations, the risk of publication bias, and the degree of the effect. There are 3 grades, such as high, moderate, low, or very low, which will assess the quality of the evidence of each of the primary results.

### 2.14 Ethics and dissemination

We do not need to obtain ethical approval in this protocol for no patients and no animals being involved. The results will be published in journals or disseminated by electronic copies.

## Author contributions

**Conceptualization:** Rumeng Chen, Yang Gao.

**Data curation:** Rumeng Chen, Xianliang Qiu, Dongqi Zhou.

**Investigation:** Rumeng Chen, Peipei Hong.

**Methodology:** Rumeng Chen, Yang Gao.

**Software:** Rumeng Chen, Xianliang Qiu.

**Supervision:** Qiu Chen.

**Writing – original draft:** Rumeng Chen.

**Writing – review:** Qiu Chen, Yang Gao.

QC is the guarantor. All authors read, provided feedback, and approved the final manuscript.

## References

[1] Bray F Ferlay J Soerjomataram I Global cancer statistics 2018: GLOBOCAN estimates of incidence and mortality worldwide for 36 cancers in 185 countries. CA Cancer J Clin 2018;68:394–424. 30207593 10.3322/caac.21492

[2] Shiboski CH Hodgson TA Ship JA Management of salivary hypofunction during and after radiotherapy. Oral Surg Oral Med Oral Pathol Oral Radiol Endod 2007;103: Suppl: S66.e1–9. 17379158 10.1016/j.tripleo.2006.11.013

[3] Porter SR Scully C Hegarty AM An update of the etiology and management of xerostomia. Oral Surg Oral Med Oral Pathol Oral Radiol Endod 2004;97:28–46. 14716254 10.1016/j.tripleo.2003.07.010

[4] Rogers SN Ahad SA Murphy AP A structured review and theme analysis of papers published on ’quality of life’ in head and neck cancer: 2000–2005. Oral Oncol 2007;43:843–68. 17600755 10.1016/j.oraloncology.2007.02.006

[5] Berk LB Shivnani AT Small W Pathophysiology and management of radiation-induced xerostomia. J Support Oncol 2005;3:191–200. 15915820

[6] Kahn ST Johnstone PA Management of xerostomia related to radiotherapy for head and neck cancer. Oncology (Williston Park) 2005;19:1827–32. discussion 1832–4, 1837–9. 16506635

[7] Nieuw Amerongen AV Veerman EC Current therapies for xerostomia and salivary gland hypofunction associated with cancer therapies. Support Care Cancer 2003;11:226–31. 12673460 10.1007/s00520-002-0409-5

[8] Cohen AJ Menter A Hale L Acupuncture: role in comprehensive cancer care--a primer for the oncologist and review of the literature. Integr Cancer Ther 2005;4:131–43. 15911926 10.1177/1534735405276419

[9] WHO. WHO traditional medicine strategy: 2014–2023; 2013. Available at: https://www.who.int/medicines/publications/traditional/trm_strategy14_23/en/. Accessed May 5, 2020.

[10] Lin JG Chen YH The role of acupuncture in cancer supportive care. Am J Chin Med 2012;40:219–29. 22419418 10.1142/S0192415X12500176

[11] Sagar SM Acupuncture as an evidence-based option for symptom control in cancer patients. Curr Treat Options Oncol 2008;9:117–26. 18688727 10.1007/s11864-008-0063-3

[12] Braga FPF Sugaya NN Hirota SK The effect of acupuncture on salivary flow rates in patients with radiation-induced xerostomia. Minerva Stomatol 2008;57:343–8. 18784633

[13] Simcock R Fallowfield L Jenkins V Group acupuncture to relieve radiation induced xerostomia: a feasibility study. Acupunct Med 2009;27:109–13. 19734380 10.1136/aim.2009.000935

[14] O'Sullivan EM Higginson IJ Clinical effectiveness and safety of acupuncture in the treatment of irradiation-induced xerostomia in patients with head and neck cancer: a systematic review. Acupunct Med 2010;28:191–9. 21062848 10.1136/aim.2010.002733

[15] Wolff A Fox PC Porter S Established and novel approaches for the management of hyposalivation and xerostomia. Curr Pharm Des 2012;18:5515–21. 22632391 10.2174/138161212803307509

[16] Zhuang L Yang Z Zeng X The preventive and therapeutic effect of acupuncture for radiation-induced xerostomia in patients with head and neck cancer: a systematic review. Integr Cancer Ther 2013;12:197–205. 22791311 10.1177/1534735412451321

[17] Naik PN Kiran RA Yalamanchal S Acupuncture: an alternative therapy in dentistry and its possible applications. Med Acupunct 2014;26:308–14. 25538815 10.1089/acu.2014.1028 PMC4270142

[18] O’Regan D Filshie J Acupuncture and cancer. Auton Neurosci 2010;157:96–100. 20605536 10.1016/j.autneu.2010.05.001

[19] Blom M Lundeberg T Dawidson I Effects on local blood flux of acupuncture stimulation used to treat xerostomia in patients suffering from Sjögren's syndrome. J Oral Rehabil 1993;20:541–8. 10412476 10.1111/j.1365-2842.1993.tb01641.x

[20] Moher D Shamseer L Clarke M Preferred reporting items for systematic review and meta-analysis protocols (PRISMA-P) 2015 statement. Syst Rev 2015;4:5–6. 25554246 10.1186/2046-4053-4-1 PMC4320440

[21] Higgins JPT TJ, Chandler J, Cumpston M, Li T, Page MJ, Welch VA (eds.). Cochrane Handbook for Systematic Reviews of Interventions version 6.0 (updated July 2019); 2019. Available at: https://training.cochrane.org/handbook. Accessed May 3, 2020.

